# Explaining utilization of HIV prevention and testing services among university students in Mozambique: results from a mixed methods study

**DOI:** 10.1186/s12889-021-11788-4

**Published:** 2021-10-19

**Authors:** Anneka Hooft, Sarah Pfeil, Josina Mussengue, Eunice Jetha, Feng He, Sonia Jain, Sandra Manuel, Patrício V. Langa, Radhika Sundararajan

**Affiliations:** 1 Department of Emergency Medicine, University of California, San Francisco, 550 16th Street, San Francisco, CA 94143 USA; 2 Department of Emergency Medicine, Pediatric Emergency Medicine, 550 16th St, 5th Floor, San Francisco, CA 94143 USA; 3 University of California, Davis, 4860 Y Street #2500, Sacramento, CA 95817 USA; 4 Universidade Eduardo Mondlane, 3453 Avenida Julius Nyere, Maputo, Mozambique; 5 Department of Family Medicine and Public Health, University of California, San Diego, 9500 Gilman Drive, La Jolla, 92093 CA USA; 6 Center for Global Health, Weill Cornell Medicine, 402 East 67th Street, New York, NY 10065 USA; 7 Department of Emergency Medicine, Weill Cornell Medicine, 585 East 68th Street, New York, NY 10065 USA

**Keywords:** HIV prevention, University students, Higher education, Gender, Mozambique, Africa

## Abstract

**Background:**

In Mozambique, HIV infection disproportionately affects young adults, particularly women. Despite awareness and knowledge of HIV transmission, many university students have not received HIV testing and continue to engage in high-risk sexual behaviors, including inconsistent condom use. Further understanding of patterns of engagement with HIV prevention and testing is key to reducing HIV transmission in this at-risk population.

**Methods:**

This study used a sequential mixed methods approach to examine patterns of engagement and perceptions of HIV prevention and testing services among higher education students in Mozambique. Survey data were collected from a representative sample of 501 students from Universidade Eduardo Mondlane (UEM) in Maputo, Mozambique to assess the primary outcomes of 1) HIV testing within the last 12 months; and 2) condom use during last sexual encounter. We employed univariate and multivariate regression models. The survey was followed by qualitative interviews with 70 survey participants which were analyzed using an inductive, content-focused analysis to further explain and contextualize survey findings.

**Results:**

Over 85% of students reported to be sexually active, among these 74% reported condom use during their last sexual encounter, and 64.2% reported an HIV test within the past 12 months. Females were more likely to have had HIV testing in the past 12 months in comparison to their male peers (aOR 1.82, 95% CI 1.11, 2.99), but were half as likely to have used a condom with their last sexual encounter (aOR 0.52, 95% CI 0.33, 0.83), when controlling for other factors. Qualitative data suggests that these discrepancies may be explained by differential perceptions in risk and trust/mistrust, with women being more concerned about infidelity by their male partner(s) and assuming more responsibility for knowing their own serostatus. Women were also subject to negative stereotypes for possessing condoms in comparison to men, which could explain lower propensity for use.

**Conclusion:**

Given gendered differences in uptake of condom use and HIV testing, and perceived HIV risk, interventions tailored specifically to male and female students may impact engagement with HIV prevention and testing and empower informed choices about sexual behaviors.

**Supplementary Information:**

The online version contains supplementary material available at 10.1186/s12889-021-11788-4.

## Introduction/background

Though HIV incidence globally is decreasing in low -and middle- income countries, it remains a significant public health threat in Mozambique. The overall prevalence of HIV in adults in Mozambique is 13.2%, which is the eighth highest in the world [1]. As in other regions of Sub-Saharan Africa, women are disproportionately affected [2]. This discrepancy is more exaggerated among younger women (ages 15–24 years), where HIV prevalence is three times the prevalence of that of young men (9.8% vs. 3.2%, respectively) [1]. As in other regions of sub-Saharan Africa, HIV transmission is largely heterosexual [1, 3].

Most university students report being sexually active, with males more than females [4]. Condom use has been proven to be effective in the prevention of HIV transmission when used consistently and correctly [5, 6]. Despite knowledge and awareness of high-risk sexual behaviors and the potential for HIV transmission, university students are vulnerable to HIV acquisition as they have multiple sexual partners, sexual encounters while intoxicated [7], and often do not employ strategies to decrease the likelihood of HIV transmission, such as consistent condom use [4, 8, 9]. Data on HIV prevalence among university students in Mozambique are limited. One hospital-based cohort study in Maputo youth demonstrated a prevalence of approximately 5%, however, only half of participants in this study were higher education students [10].

Testing and prevention are vital to ending the HIV epidemic, as testing is a critical entry point into HIV services and to establishing care for those who test positive. In Mozambique, higher education has previously been linked to an increase in the likelihood of being HIV positive, however, other studies argue that higher education is protective, and that these populations are more likely to have been tested for HIV [1, 11, 12]. How university students negotiate complicated social dynamics while mitigating the risk of contracting HIV may be affected by a variety of factors, including gender [7, 13, 14]. Several theories have been proposed to explain variations in HIV prevalence by gender, such as perceived masculinity and societal expectations influencing male engagement with HIV services, testing, and treatment [15, 16]. Intimate partner violence perpetuated by men against women has also been shown to affect contraceptive use and HIV infection risk [17, 18]. While young women are more likely to have been tested and have awareness of their HIV status in comparison to men [19], they demonstrate reduced self-efficacy in negotiating condom use [8, 20–22].

Despite the presence of HIV testing and prevention services on college campuses in Mozambique, there is scant literature on their engagement among university students and further studies are needed in order to develop and promote the best methods for HIV prevention in this vulnerable population. In order to address these gaps in knowledge, we conducted a mixed methods study among higher education students attending Universidade Eduardo Mondlane (UEM) in Mozambique. We employed an explanatory sequential study design, where quantitative survey data were used to inform delivery of qualitative interviews and integrated to provide an enhanced understanding of the complex patterns of engagement with HIV prevention and testing among higher education students [23, 24].

## Methods

### Study setting and population

**Fig. 1:**
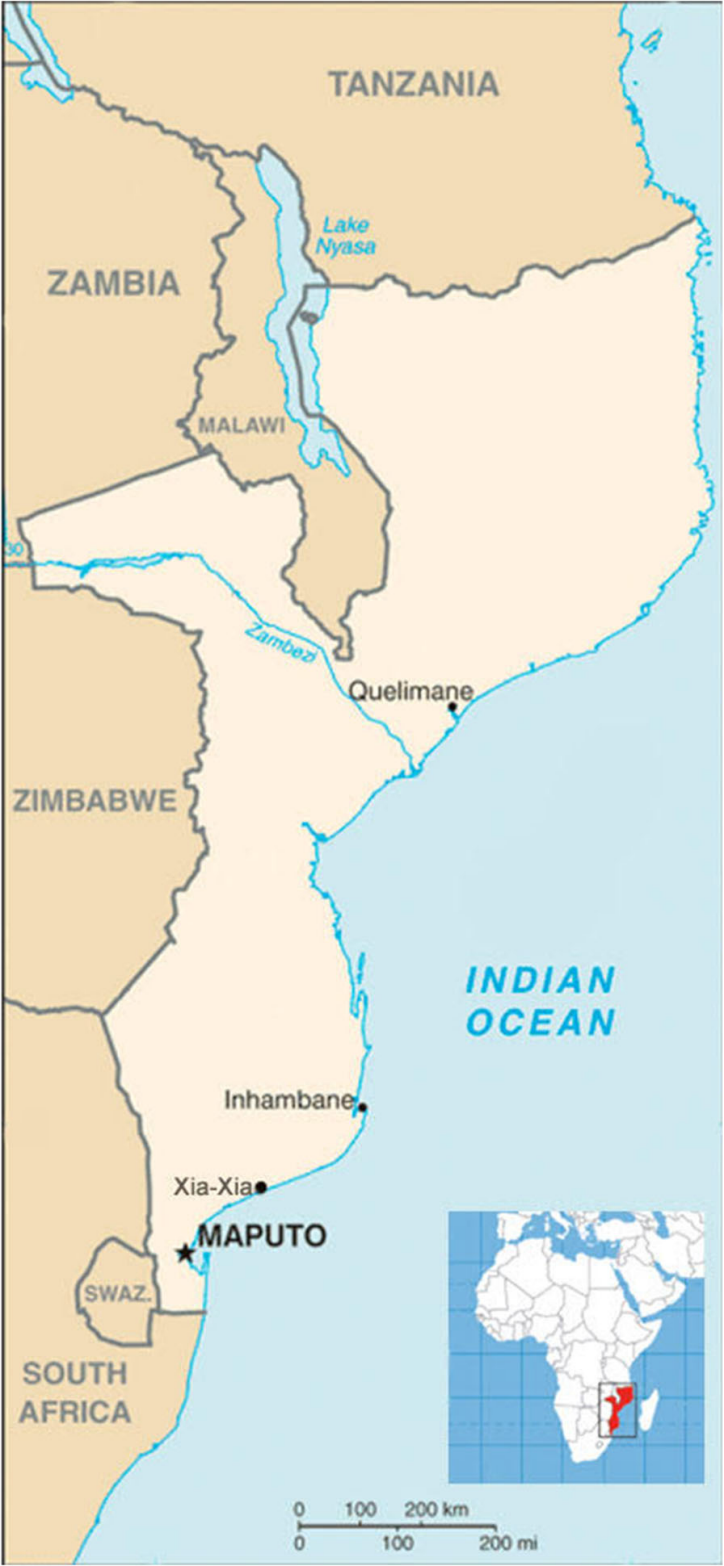
Map of Mozambique showing location of study recruitment sites (created with Adobe Photoshop 2020, version 21.1)

Mozambique is located on the Southeast coast of Africa. Maputo, the capital city, is located in the Southeast, and has a population of over one million people (see map, Fig. 1). Mozambique’s population is predominantly young, with 51% of the current population between the ages of 15–60 [25]. Universidade Eduardo Mondlane is the largest and oldest public university in Mozambique. At the time of study enrollment, approximately 26,000 students representing all 11 provinces of Mozambique were enrolled in the university. These students are distributed among four separate campuses throughout the country, and across 17 academic colleges or “Faculties”. The majority of Faculties are based in the UEM main campus in Maputo, but four Schools are located outside of Maputo city. These are the Schools of Hospitality and Rural Development (Inhambe Province), the School of Business (Gaza Province), and the School of Marine Sciences (Zambézia Province).

### Study design

**Fig. 2:**
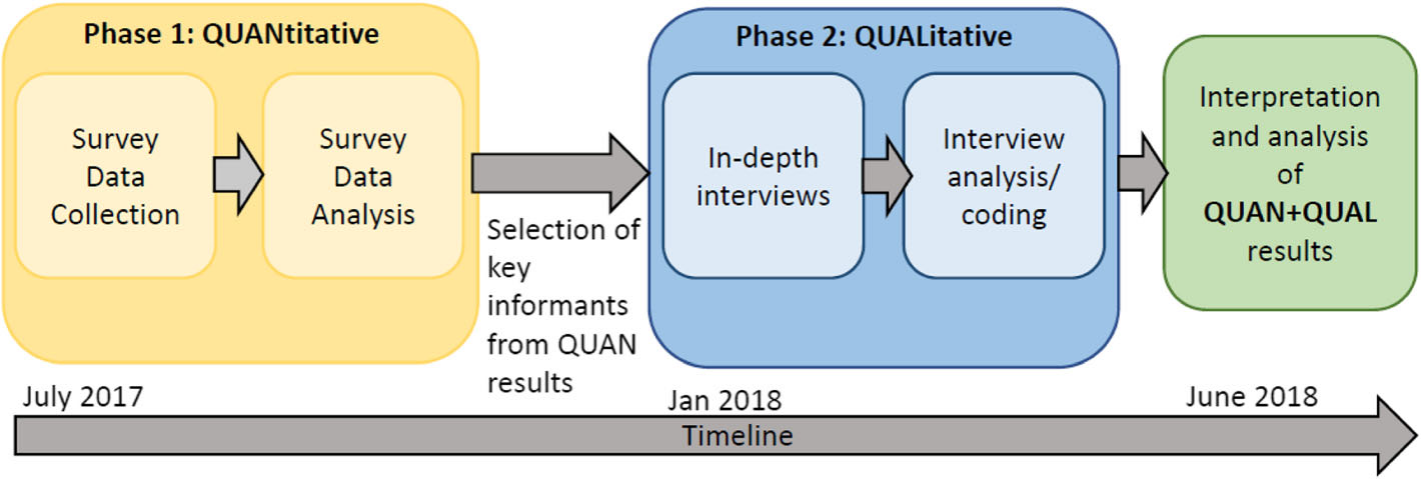
Structure and timeline of sequential mixed methods study design (based on Creswell and Plano Clark, 2017) [27]

We followed an explanatory sequential study design, with two phases of data collection, shown in Fig. 2 [26]. First, quantitative cross-sectional survey data were collected from a representative sample of students at UEM via a confidential survey questionnaire (Additional file 1). The second research phase was informed by results from the first. Based on cross-sectional results, we conducted qualitative interviews using a semi-structured interview guide (Additional file 2) with key informants to elicit contextual and granular details which could explain survey findings.

### Data collection

All data were collected between July 2017 and June 2018.

#### Quantitative sample size and recruitment

Participant recruitment was conducted at UEM between July and December 2017. A proportionate, stratified random sampling strategy was used to collect survey data from UEM students across all four campuses. Enrollment goal was *n* = 500, sampled from all 17 Faculties, representing approximately 2% of the student body. This sample size was determined based on study resources and feasibility of capturing high quality data within a six-month period. Surveys were delivered in-person by Mozambican research assistants on electronic tablets, using Open Data Kit (ODK) programming (Open Data Kit 2.0 Toolkit. 2017. URL https://opendatakit.org/). These surveys contained 75-items, including socio-demographic information, HIV testing history, condom use, relevant risk factors (such as number of sexual partners and use of commercial sex workers), and questions from the KQ-18 knowledge scale [28]. Surveys took approximately 20 min to complete.

For sampling purposes, each Faculty was considered a stratum, and students were differentially sampled from these strata to ensure proportional representation (Table 1). Using student lists provided by the UEM Registrar, students in each stratum were assigned a number, in ascending, alphabetical order, based on last name. Based on proportional survey goals for each stratum, an online random number generator (random.org) was used to identify students who would be targeted for recruitment, with males and females were recruited in proportion to student enrollment in each stratum. Students were then contacted via university email for participation in the study. If there was no response to the email invitation, an RA would call the student at the contact phone number provided to the UEM Registrar. If no response to the phone call after an additional week, a new student was randomly selected from the same stratum and gender was recruited in the same manner until our enrollment goals were met.

**Table 1:** UEM Faculties and Student enrollment estimates

Faculty Name	Number of Students (%)	Survey Goal for Stratum
Agriculture and Forestry	400 (1.5)	7
Architecture and Planning	350 (1.3)	6
Faculty of Science	5000 (19)	95
School of Law	600 (2.3)	12
Economics	500 (2)	10
Faculty of Education	6000 (23)	115
Engineering	600 (2.3)	12
Philosophy	500 (2)	10
Arts and Social Sciences	7500 (29)	145
School of Medicine	400 (1.5)	7
School of Veterinary Science	550 (2.1)	10
Communication and Arts	450 (1.7)	8
Sports Science	1200 (4.6)	24
Marine Science	300 (1.1)	6
Rural Development	250 (1)	5
Hospitality	1200 (4.6)	24
School of Business	200 (1)	5
**Total**	**26,000 (100)**	**501**

Inclusion criteria for survey participation included: 1) current enrollment at UEM; 2) no history of previously diagnosed HIV infection; 3) able to provide informed consent; and 4) age ≥ 18 years. All participants provided written, informed consent prior to participation. Students who completed surveys received 30 Mozambican Meticais cellular phone credit (~$1 USD) as remuneration for participation. At the time of enrollment in the first phase of this study, participants were advised of a possible invitation to participate in a follow up interview.

#### Qualitative sampling and recruitment

A purposive, maximum variance sampling strategy used survey results to identify key informants who could provide rich contextual data on our subjects of interest. We recruited participants who reported both frequent and rare engagement with behaviors at high risk of HIV acquisition including the following variates: 1) number of sexual partners in the last 6 months; 2) condom use during last intercourse; 3) sex with commercial sex workers; 4) number of HIV tests in the past 12 months. Attempts were also made to ensure representation of participants among age and gender. Sample size was guided by data saturation (DS), when interviews and observations no longer revealed any new or significant information [29].

Between January and June 2018, survey participants were selected as key informants and contacted by to participate in a follow up interview by a research assistant of the same gender. In-person interviews were conducted in Portuguese in private locations on the UEM campus and lasted approximately 30 min. Participants received 30 Meticais cellular phone credit (~$1 USD) as remuneration.

Six Mozambican research assistants conducted all interviews, three male and three female. These assistants had completed undergraduate degrees in Sociology, and all were trained in qualitative methods and ethics of human subjects research. Interviews were conducted, audio recorded, and transcribed in Portuguese within 72 h of completion. One research assistant fluent in both Portuguese and English was responsible for translating Portuguese into English transcripts. English transcripts were sent at the end of each week to two authors (PVL and RS) for review. PVL compared Portuguese with corresponding English translations to ensure there was consistency of meaning. Every 2 weeks, three authors (PVL, SM, RS) discussed the content of the transcripts and compared to what had already been gathered until a consensus was reached by the investigators that DS had been achieved. Enrollment was continued until 70 qualitative interviews were completed.

### Data analysis

#### Statistical analysis

Survey data were aggregated and collected into ODK and then analyzed by two authors (SJ and FH). Continuous outcomes (i.e., age, KQ-18 score) were analyzed using Wilcoxon sum tests. Fisher exact tests were used to assess associations between outcomes of interest and categorical variables such as gender or district of origin. KQ-18 scores were examined to assess for trends in variation of HIV knowledge with regard to demographic variables. Primary outcomes for this analysis were defined as 1) HIV testing within the last 12 months and 2) condom use during last sexual encounter [30]. Multivariable models for primary outcomes were created using a priori selection based on prior literature and clinical relevance, using significance of variables on frequency and univariate analysis at alpha = 0.05. Models included the following independent variables: age, gender, and number of sexual partners. All data were analyzed using the statistical software R (version 3.3.3.) (http://www.r-project.org) [31].

#### Qualitative analysis

Qualitative analysis was performed to understand participant’s lived experiences and beliefs about HIV prevention, acquisition and transmission. Using an interpretive phenomenological approach to analysis [32, 33], transcripts were initially coded for themes by the first author (AH) using an inductive, content-focused analysis through inductive, iterative engagement with the data [34, 35]. Coded text was then reviewed by authors RS, SM and PVL. A final version of codes was developed via consensus by the four researchers as DS was reached, and the entire set of transcripts was reviewed and coded using this final set of codes. Based on these codes, categories were developed pertaining to the knowledge and attitudes regarding HIV among higher education students. Categories were revised, elaborated and validated through data triangulation, via reference to multiple observation and interview sources [36]. Categories were then integrated with survey data to further explain and identify social and contextual factors that explain uptake of HIV prevention and testing.

### Ethical considerations

The study was approved by the Institutional Review Boards at both Universidad Eduardo Mondlane (Comité Institucional de Bioética para Saúde da Faculdada de Medicine/Hospital Central de Maputo) and University of California, San Diego (Human Research Protections Program, protocol # 170629).

## Results

### Survey results

We met our target enrollment goal and recruited 501 participants into the study. Overall, 530 email survey invitations were sent and 501 participants completed the survey instrument (95% response rate). Characteristics of study participants are shown in Table 2. Approximately half (*n* = 254, 50.7%) were male with an average age of ~ 23 years. The majority of students reported that they were sexually active (*n* = 430; 85.8%) with males significantly more likely to be sexually active than females (*n* = 234; 92.1% of males vs. *n* = 196; 79.4% of females, *p* < 0.001). Average age of sexual debut for both genders was 17 years. Of sexually active students, 74% (*n* = 319) reported that they used a condom the last time they had penetrative intercourse. This frequency was significantly lower in women than in men (*n* = 134; 68.4% in women and *n* = 185; 79.1%, in men, *p* = 0.015). A similar percentage of students reported having an HIV test performed in the last 12 months (*n* = 276; 64.2%), with this frequency lower in males than females (*n* = 121; 66.5% in males, and *n* = 139; 77.6% in females, *p* = 0.019).

**Table 2:** Participant Characteristics. Frequency data from self-reported survey responses from UEM student-participants

Total Participants *N* = 501			
Characteristic	Total, n (%)	Male, n (%)	Female, n (%)
**Gender**	501 (100)	254 (51)	247 (49)
**Age in years, mean (SD)**	22.7 (4.2)	23.2 (4.1)	22.1 (4.1)
**Region of Origin**			
Northern	61 (12.2)	39 (15.4)	22 (8.9)
Central	41 (8.2)	30 (11.8)	11 (4.4)
Southern (excl. Maputo)	59 (11.8)	36 (14.1)	23 (9.3)
Maputo	335 (66.9)	145 (57.1)	190 (76.9)
Other	28 (5.6)	20 (7.8)	8 (3.2)
**Religion**			
Catholic	198 (39.5)	95 (37.4)	103 (41.7)
Evangelical	125 (25.0)	52 (20.5)	73 (29.6)
Protestant	42 (8.4)	22 (8.7)	20 (8.1)
Muslim	31 (6.2)	16 (6.3)	15 (6.1)
Pentecostal	11 (2.2)	7 (2.8)	4 (1.6)
Other	65 (13.0)	36 (14.2)	29 (11.7)
None	29 (5.8)	26 (10.2)	3 (1.2)
**Marital status**			
Single	471 (94.0)	243 (95.7)	228 (92.3)
Married	30 (6.0)	11 (4.3)	19 (7.7)
**Ever sexually active**	430 (85.8)	234 (92.1)	196 (79.4)
**Sexual identity**			
Heterosexual	410 (81.8)	219 (86.2)	191 (77.3)
Homosexual	13 (2.6)	11 (4.3)	2 (0.8)
No history of relationships	77 (15.4)	24 (9.5)	53 (21.5)
Other	1 (0.2)	0 (0)	1 (0.4)
**Number of sexual partners in last 6 months, mean (SD)**	1.6 (2.0)	1.9 (2.6)	1.1 (0.8)
**Used a condom with last sexual encounter, n (% sexually active participants)**	319 (74.2)	185 (79.1)	134 (68.4)
**Ever had an HIV test**	398 (79.4)	193 (76.0)	205 (83.0)
If yes, number of lifetime HIV tests			
1		54 (28.0)	50 (24.3)
2–4		93 (48.1)	87 (42.4)
> 4		46 (23.8)	68 (33.1)
**Had an HIV test within past 12 months, n (% sexually active participants)**	276 (64.2)	127 (54.3)	149 (76.0)
**Have utilized sex workers for sex, n (% sexually active participants)**	41 (9.5)	41 (17.5)	0 (0)
**K-18 score, mean (SD)**	12.7 (3.0)	12.8 (3.1)	12.7 (2.9)

#### Predictors of HIV testing and prevention services utilization

Among sexually active students, females were three times more likely than males to have ever received an HIV test after adjusting for co-variates (aOR = 3.38; 95% CI 1.84–6.23, *p* < 0.001). Females were also slightly more likely to have received an HIV test in the past 12 months when compared to males (aOR 1.82; CI 1.1–3.0, *p* = 0.017) (Table 3). With regard to use of condoms for HIV prevention, multivariable analysis demonstrated that female students were half as likely (aOR = 0.52; 95% CI 0.33–0.83, *p* = 0.006) to have used a condom during their last sexual encounter when compared to male students (Table 4). Age was also significant in predicting condom use, with every one-year increase in age decreasing odds of condom use by 0.1 (aOR 0.9; 95% CI 0.86–0.95; p < 0.001) (Table 4).

**Table 3:** Variables associated with HIV testing in the past 12 months, univariate, and multivariate regression models. Multivariate outcomes adjusted for number of sexual partners, gender, condom use with last sexual intercourse, and age

Variable	N (%) or median (IQR) ^€^		***p***-value (χ^**2**^)	OR (95% CI)	p-value	aOR (95% CI)	p-value
HIV Test	Yes	No					
**Gender**							
Male	121 (66.5)	61 (33.5)	0.019*	referent	0.018*	referent	
Female	139 (77.6)	40 (22.4)		1.75 (1.10–2.80)		1.83 (1.11–2.99)	0.017*
**Condom at last intercourse**							
No	69 (74.2)	24 (25.8)		Referent			
Yes	191 (71.3)	77 (28.7)	0.688	0.86 (0.51–1.47)	0.588	n/a	
**Number of Sex Partners**	1 (1–2)	1 (1–2)	0.480	1.02 (0.83–1.24)	0.872	n/a	
**Age**	22.5 (21–25)	22 (21–25)	0.858	0.99 (0.94–1.04)	0.678	1.00 (0.94–1.05)	0.853

*significant at *p* = 0.05 level

^€^n(%) for categorical variables and median (IQR) for continuous variables

**Table 4:** Variables associated with condom use with last sexual encounter among sexually active participants, univariate and multivariate regression models. Multitvariate outcomes adjusted for number of sexual partners, gender, and age

Variable	N (%) or median (IQR) ^€^		***p***-value (χ^**2**^)	OR (95% CI)	***p***-value	aOR (95% CI)	***p***-value
Condom at last intercourse	Yes	No					
**Gender**							
Male	185 (79.1)	49 (20.9)	0.015*	referent		referent	
Female	134 (68.4)	62 (31.6)		0.57 (0.37–0.89)	0.012*	0.52 (0.33–0.83)	0.017*
**Number of Sex Partners**	1 (1–2)	1 (1–2)	0.283	1.03 (0.90–1.18)	0.651	n/a	
**Age**	22 (20–24)	23 (21–26)	< 0.001*	0.91 (0.87–0.96)	< 0.001*	0.91 (0.86 = 0.95)	< 0.001*

*significant at *p* = 0.05 level

^€^n (%) for categorical variables and median (IQR) for continuous variables

### Qualitative results

There were 70 total interview participants, 49 of whom were male and 21 of whom were female, with a mean age of 22.7 years (SD ±5.5 years). All 70 survey participants approached for the qualitative study phase agreed to participate in an interview. Two major themes emerged to explain uptake of HIV testing and condom use among our participants: 1) risk perception and 2) trust/mistrust. These themes explain differential engagement with HIV testing and condom use among female and male students pertinent to the significant survey findings. We present representative quotes from the qualitative interviews to explain patterns of HIV prevention and testing utilization in Table 5 and summarize our findings below.

**Table 5:** Integration of quantitative and qualitative study results showing quantitative survey result and representative quotes from qualitative interviews explaining the finding

**Quantitative result: Females use condoms less frequently than males**	
Qualitative explanation: Gender norms support men to decide whether condoms will be used, and women who carry condoms are perceived as untrustworthy.	
“We men get very upset when we find condoms in our partners’ bags, but I think everyone should walk with a condom. But it’s annoying because [if] you stay in that, that this one is not safe, at any moment she can use [the condom].” (Male, age 30s)	
“I don’t use [condoms] because my partner doesn’t accept them. [The condom], it’s mine, I always have them. He doesn’t accept [using] it… …[so] I don’t use it” (Female, Age 20s)	
**Quantitative result: Women are three times more likely to have received an HIV test**	
Qualitative explanation: Greater perceived risk among women motivates increased utilization of HIV testing.	
“I found out that [HIV is] not really [a concern for me]. HIV doesn’t kill you, understand? It changes the person’s lifestyle… …I don’t see any problem in that. All right, it gives a problem, a disease that changes your lifestyle… …if the person has it can assume the will, and sometimes even believe, right? There is no fear of HIV. It’s the fear of getting pregnant… and more out there.” (Male, Age 20s) “I think [I am at risk of contracting HIV]. It’s not because it may not be on my part, but I have my partner. He can go out, contract the disease and pass it to me. This is the fear I really have. I can’t say that he is faithful to me totally because it’s hard to say, I don’t always go out with him.” (Female, Age 20s)	
**Quantitative result: Consistent condom use declines with age**	
Qualitative explanation: Condoms are not used in the context of committed, stable relationships where partners trust one another.	
“They say [the] condom isn’t fun. The relationship made with [a] condom isn’t fun. First, others say [it is] because they trust their partner. And the ones [who] use say they used [a condom] because they don’t know the person yet, or don’t have that intimacy with that person. I think after a while the person [who] says he takes it, no longer uses it.” (Male, Age 30s) “I didn’t use [a condom]. There are many reasons. It’s because now, right, by chance, I happen to be living with my partner, so [we have] that trust that exists between two people. The only method I use for now is the pill… …for example this year, I won’t lie I’ve never used [a condom].” (Female, Age 20s)	

#### Women are three times more likely to receive HIV testing because they have higher perception of HIV risk

Though both male and female students perceive themselves at some risk of contracting HIV, men demonstrate more nonchalance toward the disease and less concern about knowing their serostatus. Other potential ways of contracting HIV, such as using shared instruments at a barbershop, were frequently mentioned in interviews, as if to decrease the emphasis on personal responsibility for high risk behavior.

*“There is another factor [in HIV transmission] … we use the same hairdressing salons. So, there is a probability of anything happening, but not through sexual pathways.”* (Male, Age 20s)

One participant describes why he does not feel that HIV is a concern for him, rather he is more concerned about unwanted pregnancy “and more”:

*“HIV doesn’t kill you, understand? It changes the person's lifestyle … I don’t see any problem in that. All right, it gives a problem, a disease that changes your lifestyle … There is no fear of HIV [with intercourse]. It’s the fear of getting pregnant… and more out there.”* (Male, Age 20s)

In contrast to the perspective of men, women acknowledge the importance of testing in order to know their status to protect their health and potentially start treatment for HIV as early as possible for the best outcome.

*“I don’t want any disease, and mainly HIV/AIDS, so I do everything to prevent myself [from getting it]. And I haven’t always used condoms, but at least I do the test in case I find out soon to try a way to start treatment*.” (Female, Age 30s)

Other women report they are not personally engaging in high risk behaviors, but they assume increased personal HIV transmission risk related to the behaviors of male partners. They perceive themselves at elevated risk because the sexual behaviors of their male partners are unknown:

*“I think [I am at risk of contracting HIV]. It's not because it may not be on my part, but I have my partner. He can go out, contract the disease and pass it to me. This is the fear I really have. I can’t say that he is faithful to me totally because it's hard to say. I don’t always go out with him.”* (Female, Age 20s)

#### Females use condoms less frequently than males because condoms are linked to intimate partner mistrust

Though some participants feel the responsibility for condom use is shared by both members of a relationship, in practice, the decision to use or not use a condom during intercourse is often described as a decision to be made by the male. Females describe deferring to the male’s preference regarding whether a condom is used.

*“Normally, [using a condom], it’s already established that [it is the] idea of ​​a man. If the man doesn’t put it [on], then the woman lets it pass”.* (Female, Age 20s)

*“I don’t use [condoms] because my partner doesn’t accept them. [The condom] it's mine, I always have them. He doesn’t accept [using] it … [so] I don’t use it.”* (Female, Age <20)

In addition, women are perceived as untrustworthy if they have condoms with them.

*“Maybe for cultural reasons, it’s up to the man [to provide the condom]. Come on, it’s kind of bizarre to find women with condoms. Not because they shouldn’t, [or] they can’t, but [because of] cultural issues, habits, customs that we have. Who usually has [a condom] is the man.* (Male, Age 20s)

Specifically, men perceive condoms as representing opportunity for a female partner to have sexual relationships with other men. One participant questioned the “safety” of being in a relationship with a woman who carries her own condoms:

*“We men get very upset when we find condoms in our partners' bags, though I think everyone should walk with a condom. But, it's annoying because [if] you stay in that [relationship, you may think to yourself], ‘this one is not safe, at any moment she can use [the condom].’*” (Male, Age 30s)

#### Consistent condom use declines with age as trust increases within committed relationships

Commitment to a relationship and the level of trust between two people also influences whether or not a couple chooses to use a condom during intercourse. Condoms are described as a barrier to intimacy between partners and are considered more acceptable in relationships where partners are less familiar with one another. Over time, partners build trust that the relationship is monogamous. With increased trust, they perceive less risk of HIV acquisition with unprotected sexual intercourse.

*“They say [the] condom isn’t fun. The relationship made with [a] condom isn’t fun. First, others say [it is] because they trust their partner. And the ones [who] use [a condom] say they used it because they don’t know the person yet, or don’t have that intimacy with that person”* (Male, Age 30s)

Our participants describe how condom use is uncommon between committed partners. In fact, it is a marker of trust to not use a condom during intercourse with your regular partner.

*“Most of the people I talk to say, ‘with my boyfriend, with my girlfriend, I don’t use a condom.’”* (Male, Age 30s)

Both males and females discussed that condom use overall decreases the longer a couple has been together, and the likelihood of being in a committed relationship increases with age.

*“I didn’t use [a condom]. There are many reasons. It's because now, right, by chance, I happen to be living with my partner, so [we have] that trust that exists between two people. The only method I use for now is the pill … for example this year, I won’t lie I’ve never used [a condom].”* (Female, Age 20s)

## Discussion

This study examines high risk behaviors for HIV acquisition in a population of university students using qualitative data to provide descriptive context for results from a cross-sectional survey. We found that one-quarter of sexually active students did not use a condom during their last sexual encounter. This was even lower among females, with nearly 1/3 of whom did not use a condom. Only about half of all students reported having undergone recent HIV testing. We found significant variation in HIV testing by gender, with female students with much higher odds of having HIV testing, but with much lower odds of condom use when controlling for other factors. Our qualitative data describes how variation in perceived risk of contracting HIV and trust/mistrust by gender impacts uptake of HIV prevention and testing resources.

Our findings are consistent with previous studies indicating that condom use is generally lower in young women versus young men and within established, trusting relationships [4, 10, 11, 37–40], including longer term, monogamous relationships, where family planning considerations may also influence the decision to use condoms. Our results, along with others, also indicate that fear of pregnancy dominates the selection of birth control method, and condoms may be considered only one among many methods to avert pregnancy, rather than primarily being considered for promotion of good health or decreasing HIV risk [4, 41]. Pressure on females by males to forgo condom use for a more pleasurable or intimate sexual experience in a trusting relationship has also been shown to alter high risk behavior [41]. Our finding that condom use also decreases with age may be a proxy for being in a more “trusting” long-term relationship, but further research is needed to evaluate this potential association.

Variation in HIV testing behavior by gender in university students has been previously demonstrated, though not as consistently [42, 43]. Our qualitative results indicate that there are fundamental differences in perceived risk of contracting HIV and fear of the disease itself in males versus females. Though participants of both genders often describe engaging in risky behaviors knowing that this increases their likelihood of contracting HIV, males seem to be more comfortable with this risk, or felt it was out of their control given a number of potential factors that may contribute to HIV transmission. Females on the other hand, have more concern that even when they do not personally engage in risky sexual behavior, their partner may. They assume the HIV risk of their partner, which motivates them to be more proactive to know their own status. These findings are consistent with prior studies demonstrating that females have higher rates of engagement with heath care and preventive services [44–47].

These variations in high-risk behavior may present new targets for HIV education and prevention in this population that can be tailored in a gender-specific way to address barriers to HIV prevention that we have identified. These may include female empowerment programs, such as the ongoing [27], that focus on the desire to protect oneself, which an important motivating concept underlying health engagement among women. Additional strategies may include campaigns focused on men that normalize the idea of women having access and using condoms, or HIV testing outreach focused on reducing stigma among gender-specific peer groups [48, 49]. These strategies are also relevant to youth populations outside of higher educations, who may be at higher risk of HIV acquisition and transmission.

Limitations of this study included a lack of assessment for pre-exposure prophylaxis (PrEP) at HIV prevention, as the study was conducted prior to widespread rollout of PrEP in Mozambique. Information was self-reported and HIV serostatus was not verified prior to inclusion/exclusion from the study. The study recruitment population was limited to a single university in a large city in Mozambique, however, every effort was made to get a representative sample of students of both genders within this institution.

## Conclusion

University students in HIV-endemic areas are at high risk for contracting HIV due to their unique social situation and peer influence on their behavior and sexual practices. There are gendered differences in condom use, HIV testing, and perceived HIV risk. Interventions tailored specifically to young males and females in these environments may improve educational reach and empower students to make informed choices about sexual behaviors.

## Supplementary Information

**Additional file 1.** Survey Questionaire. Survey questions administered to UEM students.

**Additional file 2.** Semi-structured Interview Guide. Interview guide used to collect qualitative data from UEM students.

## Data Availability

The datasets used and/or analysed during the current study are available from the corresponding author on reasonable request.

## Abbreviations

UEM: Universidade Eduardo Mondlane
ODK: Open Data Kit
DS: Data saturation
