# Supplementary material for: Explaining utilization of HIV prevention and testing services among university students in Mozambique: results from a mixed methods study
Source: BMC Public Health. 2021 Oct 19;21:1891. doi: 10.1186/s12889-021-11788-4 (PMC8525004; doi:10.1186/s12889-021-11788-4)
Supplement: Supplementary file 1 — Additional file 1. Survey Questionaire. Survey questions administered to UEM students. [file 12889_2021_11788_MOESM1_ESM.docx]

**Socio-demographic characteristics:**

1. How old are you?
2. Sex
   1. Male
   2. Female
3. Marital status
   1. Single
   2. Married
   3. Separate
   4. Widowed
4. I have sexual relations with
   1. Men
   2. Women
   3. Both
   4. Never had sexual relations
5. Religion
   1. Catholic
   2. Muslim
   3. Protestant
   4. Pentecostal
   5. Evangelical
   6. Hindu
   7. None
   8. Other
6. What province are you from?
   1. Cabo Delgado
   2. Niassa
   3. Zamezia
   4. Nampula
   5. Tete
   6. Manica
   7. Sofala
   8. Gaza
   9. Inhambane
   10. Maputo
   11. Other
7. What is your academic major?
8. Which year are you in?
   1. First
   2. Second
   3. Third
   4. Fourth
   5. Fifth
   6. Sixth
   7. Other
9. Do you have a scholarhip to attend UEM?
   1. Yes
   2. No
10. If you are on scholarship, which residence hall do you reside in?
    1. R1
    2. R4
    3. R7
    4. R8
    5. I do not live at the university campus
11. How old were you when you had first sexual intercourse?
    1. Age ___
    2. Never had intercourse
12. If you have had sexual intercourse previously, when was the last time you had intercourse?
    1. Less than a week ago
    2. Between a week and a month
    3. Between 1-3 months
    4. More than 3 months
13. Did you use a condom last time you had sexual intercourse?
    1. Yes
    2. No
14. If yes to Q13, what was the main reason that you used a condom (choose one)?
    1. I wanted to prevent HIV
    2. I wanted to prevent pregnancy
    3. I wanted to prevent both HIV/pregnancy
    4. I did not trust my partner
    5. My partner asked/ insisted
    6. Other
    7. I do not know
15. If no, to Q13, what was the main reason you didn’t use a condom (choose one)?
    1. I never use them
    2. I did not have one
    3. I was under the influence of alcohol/drugs
    4. My partner refused
    5. I trust my partner
    6. I would only use it with prostitutes
    7. I did not have the courage to ask my partner
    8. I do not trust the condom
    9. I am not scared to get HIV or STI
    10. I was excited and ignored the risk
    11. I wanted to have a kid
16. The last time you used a condom during intercourse whose idea was it?
    1. Mine
    2. Partners
    3. Both
17. If your partner refuses to use a condom during sexual intercourse, what do you do?
    1. I accept their decision and we have unprotected sex
    2. I will try to convince them to use a condom, but might have unprotected sex
    3. I do not have sex without a condom
18. How many sexual partners have you had intercourse with in the last six months?
    1. __
19. Have you ever had sexual intercourse with commercial sex workers?
    1. Yes
    2. No
20. If yes to Q19, in the last 12 months, have you had intercourse with a commercial sex worker?
    1. Yes
    2. No
21. If yes to Q19, in those sexual relationships did you use a condom?
    1. Always
    2. Sometimes
    3. Never
22. Are you currently using any strategies to prevent HIV/AIDS?
    1. Monogamy/ faithful to a partner
    2. Abstinence
    3. Yes, Other
    4. No
23. Has your knowledge of HIV/AIDS influenced your sexual behavior?
    1. Yes
    2. No
24. If yes to Q23, in what way has knowledge of HIV/AIDS influenced your sexual behavior (select all that apply)?
    1. I stopped having sex completely
    2. I started using a condom with intercourse
    3. I only have one sexual partner
    4. I reduced my number of sexual partners
    5. Other
25. If answer B to Q11, what is the main reason you’ve never had sexual intercourse?
    1. I am too young
    2. I have not found the right person yet
    3. I am waiting until marriage
    4. I am scared of getting pregnant
    5. I am scared of getting an STI
    6. Other
    7. I do not know
26. How have you learned about HIV/AIDS (please answer yes or no for the below)?
    1. Radio
    2. Television
    3. Newspaper/magazines
    4. Pamphlets
    5. Billboard
    6. Church
    7. University
    8. Activists at the university
    9. Professors at the university
    10. Friends
    11. Family
    12. Local public health clinic
    13. Private health clinic
    14. Teenage health clinic
    15. Telephone
    16. Other
27. Do you think HIV has a cure?
    1. Yes
    2. No
    3. I do not know
28. If yes to Q27, how do you believe can you cure HIV (answer yes or no for all the answer choices):
    1. Sexual intercourse with a child/virgin
    2. Medications
    3. Traditional medicines
    4. Antiretroviral therapies
    5. Other
29. Do you know how you can avoid HIV?
    1. Yes
    2. No
    3. I do not know
30. If yes to Q29, how can you avoid HIV/AIDS (Answer yes or no for all of following)?
    1. Do not have intercourse
    2. Always use a condom
    3. Only have 1 sexual partner
    4. Lower the number of sexual partners you have
    5. Faithfulness
    6. Coitus interruptus
    7. Only have sexual intercourse with your married partner
    8. Have intercourse with a virgin
    9. Avoid vaccines
    10. Do not share razors
    11. Do not eat with HIV-infected people
    12. Do not have sex with homosexuals
    13. Avoid blood transfusions
    14. Do not give blood
    15. Avoid contact with fluids
    16. Only use disposable/ sterilized needles and syringes
    17. Avoid kissing in the mouth
    18. Do not socialize with HIV-infected people
    19. Do not have intercourse with sex workers
    20. Do not share bathrooms
    21. Do not use the pool
    22. Other
    23. I do not know
31. With whom do you think it’s acceptable to talk about HIV? (answer yes or no to each of the following)
    1. Parents
    2. Siblings
    3. Other family members
    4. Partner
    5. Colleagues
    6. Professors
    7. Local activists
    8. Priest
    9. Doctors/nurses
    10. Traditional healers
32. Do you think your personal risk of acquiring HIV is little, moderate, high, or are you at no risk at all?
    1. No risk
    2. Little risk
    3. Moderate risk
    4. High risk
    5. I do not know
33. If A or B to Q32, why do you think you have little to no risk of contracting HIV? (answer yes or no for each of the following)
    1. Never injected drugs
    2. I do not have sexual intercourse
    3. I always use a condom
    4. I have limited number of sexual partners
    5. My partner doesn’t have other partners
    6. I have never received a transfusion of blood
    7. I only use disposable/sterile syringes and needles
    8. I do not share objects that can cut you such as razors
    9. I trust my partner
    10. I am careful with fluids of others
    11. Other
34. If C or D to Q33, Why do you think you have a moderate/ high risk (answer yes or no for each of the following)
    1. I use / inject drugs
    2. I do not use a condom
    3. I have more than one sexual partner
    4. My sexual partner has other sexual partners
    5. I have received blood transfusions
    6. I do not use disposable/sterile syringes and needles
    7. I share sharp objects such as razors
    8. I usually have sex when I am drunk
    9. I am not careful with other people’s fluids
    10. Other

For the next 18 questions*, please answer if the statement is true, false, or if you don’t know the answer.

1. Pulling out the penis before a man climaxes/ejaculates keeps that partner from getting HIV during sex:
   1. True
   2. False
   3. I do not know
2. Someone can get HIV by sharing a glass of water with someone who is HIV infected:
   1. True
   2. False
   3. I do not know
3. A woman can get HIV through anal sex with a man:
   1. True
   2. False
   3. I do not know
4. Showering or washing one’s genitals after sex can prevent that person from getting HIV:
   1. True
   2. False
   3. I do not know
5. Coughing and sneezing DO NOT spread HIV:
   1. True
   2. False
   3. I do not know
6. All pregnant women infected with HIV will have babies born with AIDS:
   1. True
   2. False
   3. I do not know
7. People with HIV quickly show signs of being infected:
   1. True
   2. False
   3. I do not know
8. There is a vaccine that can prevent HIV infection
   1. True
   2. False
   3. I do not know
9. You can get HIV by deep kissing, putting your tongue in your partner’s mouth, if your partners has HIV:
   1. True
   2. False
   3. I do not know
10. A woman cannot get HIV if she has sex during her menstrual period:
    1. True
    2. False
    3. I do not know
11. There is a female condom that can decrease a woman’s risk of getting HIV through sex:
    1. True
    2. False
    3. I do not know
12. A natural skin condom works better against protecting from HIV transmission than latex condoms:
    1. True
    2. False
    3. I do not know
13. A person WILL NOT get HIV if he or she is taking antibiotic medication:
    1. True
    2. False
    3. I do not know
14. Having sex with more than one partner can increase a person’s chance of getting HIV:
    1. True
    2. False
    3. I do not know
15. A person can get HIV by swimming in a hot tub or swimming pool with a person with HIV:
    1. True
    2. False
    3. I do not know
16. A person can get HIV through oral sex:
    1. True
    2. False
    3. I do not know
17. Taking a test for HIV one week after having sex will tell if a person has been infected with HIV:
    1. True
    2. False
    3. I do not know
18. Using oil with condoms lowers the risk of getting HIV during sex:
    1. True
    2. False
    3. I do not know
19. Do you believe that HIV and AIDS the same thing?
    1. Yes
    2. No
    3. I do not know
20. Does eating healthy foods prevent your risk of contracting HIV/AIDS?
    1. Yes
    2. No
    3. I do not know
21. Can a person get HIV/AIDS if they are bewitched?
    1. Yes
    2. No
    3. I do not know
22. Can a person with HIV take medications to prevent being sick with AIDS?
    1. Yes
    2. No
    3. I do not know
23. Do you think a traditional healer can protect you from contracting HIV/AIDS?
    1. Yes
    2. No
    3. I do not know
24. Does a person with an HIV have a greater chance of contracting AIDS?
    1. Yes
    2. No
    3. I do not know
25. Can a person contract HIV/AIDS through a mosquito bite?
    1. Yes
    2. No
    3. I do not know
26. Can HIV/AIDS be transmitted from a mom to their child (please choose yes or know for each of the following)?
    1. During pregnancy
    2. During birth
    3. During breastfeeding
27. Do you know who is living with HIV or who has died because of HIV/AIDS?
    1. Yes
    2. No
28. If you knew that a worker at a farmers market had HIV/AIDS would you buy their products?
    1. Yes
    2. No
    3. I do not know
29. If a person in your family had HIV/AIDS, would you want to keep it a secret?
    1. Yes
    2. No
    3. I do not know
30. If a person in your family had HIV/AIDS, would you be willing to take care of them at your house?
    1. Yes
    2. No
    3. I do not know
31. If one of your professors had HIV/AIDS but weren’t sick, do you think they could still teach at the university?
    1. Yes
    2. No
    3. I do not know
32. If one of your colleagues had HIV, would you be willing to share a room as them?
    1. Yes
    2. No
    3. I do not know
33. Have you heard of counseling and voluntary HIV testing?
    1. Yes
    2. No
    3. I do not know
34. Do you know where you can get an HIV test?
    1. Yes
    2. No
    3. I do not know
35. If yes to Q68, where can you go to get an HIV tet?
    1. Health clinic
    2. Pharmacy
    3. Traditional healer
    4. Other
36. Have you ever had an HIV test?
    1. Yes
    2. No
37. If yes to Q70, how many test have you received in your lifetime?
    1. 1
    2. 2
    3. 3
    4. 4
    5. More than 4
38. If yes to Q70, When was the last time you had an HIV test?
    1. Less than 3 months ago
    2. 3-12 months ago
    3. More than 12 months ago
39. If yes to Q70, did you receive counseling the last time you had an HIV test?
    1. Yes
    2. No
40. If yes to Q70, what was the main reason for your last HIV test (select one)?
    1. I wanted to know my status
    2. The doctor recommended it
    3. My partner tested positive
    4. My partner asked
    5. I wanted to get married
    6. I wanted to get pregnant
    7. Because of my job
    8. For a scholarship
    9. To study abroad
    10. For insurance
    11. To get a loan from the bank
    12. I have a life that is full of risks
    13. Other
41. If no to Q70, what is the main reason you have you never received an HIV test (select one)?
    1. I’m scared to know if I’m positive
    2. I’m scared of the stigma of HIV
    3. I do not trust the confidentiality of the testing process

*Questions 35-52 from Carey, M. P., & Schroder, K. E. E. (2002). Development and psychometric evaluation of the brief HIV knowledge questionnaire (HIV-KQ-18). *AIDS Education and Prevention*, 14, 174-184.
