# Supplementary material for: Explaining utilization of HIV prevention and testing services among university students in Mozambique: results from a mixed methods study
Source: BMC Public Health. 2021 Oct 19;21:1891. doi: 10.1186/s12889-021-11788-4 (PMC8525004; doi:10.1186/s12889-021-11788-4)
Supplement: Supplementary file 2 — Additional file 2. Semi-structured Interview Guide. Interview guide used to collect qualitative data from UEM students. [file 12889_2021_11788_MOESM2_ESM.docx]

HIV knowledge among UEM students

Qualitative Interview Guide

Participant ID number:

Date of interview:

Name of interviewer:

Thank you for taking the time to speak with me. Please remember that everything we discuss is confidential, and will not be shared with anyone outside the research team. We hope you will share your honest experiences so that we can learn how to provide better health and preventative services to the UEM student community.

For participants who are sexually active:

*1. Sexual Debut and number of sexual partners*

- How old were you when you first became sexually active?

- What made you decide to become sexually active with that particular person?

-Looking back on it now, what do you think about it?

- Thinking about your past sexual encounters, can you tell me what factors have influenced you to have intercourse with someone?

(i.e., love, trust, intimacy, physical attraction, partner expectation, coercion, expectation of financial/material gain, etc)

- Thinking about your past sexual encounters, can you describe any factors that have dissuaded you from having intercourse with someone?

(i.e., lack of trust, fear of HIV, unwilling to commit to relationship, fear of rejection, etc)

- Do you think you have had more sexual partners than other students your age?

- Why or why not?

- How does that make you feel?

*2. Condom utilization*

- Was a condom utilized during the last time you had sexual intercourse?

- If yes, whose condom was it? (participant or the partners?)

- If no, please tell me more about why you did not use a condom.

- Whose responsibility is it to remember to use a condom during intercourse?

- Please tell me what you know about condoms, and why some people use them.

- Do you think most students at your university use condoms when they have sexual intercourse? Why or why not?

For participants who are NOT sexually active:

*1. Abstinence*

- Please tell me about your reasons for being abstinent.

- What are the most important factors supporting your abstinence?

- Are there any challenges to staying abstinent?

- Do you think that people of your gender are expected to be abstinent at your age? Why or why not?

- Abstinence from intercourse can mean different things to different people. Can you tell me what, if any, sexual activities you have participated in, that do not include intercourse (For example, oral sex, anal sex, or ‘heavy petting’)?

*2. Condom utilization*

- Please tell me what you know about condoms, and why some people use them.

- Do you think most students at your university use condoms when they have sexual intercourse? Why or why not?

For all participants:

*3. High risk behaviors*

- Have you ever utilized a commercial sex worker?

- if yes, please tell me more about that (i.e., when did you start? How often? Why do you use them?).

- if no, do you know of any student who use sex workers?

- What do you know about injection drug use?

- Do you use them?

- Do you know any students who do?

- What do you think about people who use IV drugs?

*4. HIV testing service engagement*

- Have you ever received an HIV test?

- if no, please tell me about why you have never previously tested for HIV

- if yes, when was your last test, and what was your result?

- Please tell me about your experience receiving that test (i.e., were you nervous? Afraid to be recognized? Where did you receive the test, and why? Did anyone accompany you?)

- Do you think most students at your university have received an HIV test in the past year? Why or why not?

- Do you consider yourself at risk for contracting HIV? Why or why not?

- Do you know anyone who is living with HIV?

- If yes, tell me about that person, and what their life is like.

- If no, what do you think life is like for someone living with HIV?

Do you have any questions for me, or would you like to add anything to what we have previously discussed?

Thank you for your time and for participating in our research study!

Version Date 19 January 2018
